# Differential Gender-Dependent Patterns of Cardiac Fibrosis and Fibroblast Phenotypes in Aging Mice

**DOI:** 10.1155/2020/8282157

**Published:** 2020-05-23

**Authors:** Angela Achkar, Youakim Saliba, Nassim Fares

**Affiliations:** Laboratoire de Recherche en Physiologie et Physiopathologie, Pôle Technologie Santé, Faculté de Médecine, Université Saint Joseph, Beirut, Lebanon

## Abstract

Aging is characterized by physiological changes within the heart leading to fibrosis and dysfunction even in individuals without underlying pathologies. Gender has been shown to influence the characteristics of cardiac aging; however, gender-dependent cardiac fibrosis occurring with age remains largely not elucidated. Thus, broadening our understanding of this phenomenon proves necessary in order to develop novel anti-fibrotic strategies in the elderly. In this study, we aim to characterize cardiac fibrosis and cardiac fibroblast (CF) populations in aged male and female mice. Echocardiography revealed eccentric hypertrophy with left ventricular dilatation in the aged male versus concentric hypertrophy with left posterior wall thickening in the female, with preserved cardiac function in both groups. Reactive fibrosis was evidenced in the myocardium and epicardium of the aged female mice hearts whereas perivascular and replacement ones where present in the male heart. Collagen I was predominant in the aged male heart whereas collagen III was the main component in the female heart. CFs in the aged male heart were mainly recruited from resident PDGFR*α*^+^ populations but not derived from epicardium as evidenced by the absence of epicardial progenitor transcription factors Tcf21, Tbx18 and Wt1. Our results present a paradigm for gender-dependent cardiac fibrosis and the origins of CFs with age. This sets forth to revisit cardiac anti-fibrotic management according to the gender in the elderly and to explore novel therapeutic targets.

## 1. Introduction

Aging is an important risk factor for cardiovascular-related morbidity and mortality. Aged heart exhibits myocardial remodelling that includes among others increased apoptosis and oxidative stress, hemodynamic changes, cardiomyocyte senescence and collagen deposition leading to cardiac fibrosis [1, 2]. Notably, aging-related cardiac fibrosis has been demonstrated in animals [3, 4] and humans [1, 5–7] even in the absence of underlying pathologies. Fibrotic tissue is stiffer and less compliant [8], resulting in subsequent cardiac dysfunction and heart failure but with normal or nearly normal ejection fraction. This is known as heart failure with preserved ejection fraction (HFpEF), the most common type of heart failure in the elderly [1, 2, 9].

Gender has been shown, clinically and in experimental models to influence the characteristics of cardiac remodelling in disease. This was associated in men with greater expression of fibrotic markers such as collagen genes that further contribute to cardiac dysfunction [10, 11]. Besides, cardiac imaging revealed distinct profiles of cardiac fibrosis in elder men and women [5, 7, 12]. Till date, there is no direct evidence whether age-related cardiac fibrosis is differential between the male and female.

CFs are the main actors responsible of cardiac fibrosis [13, 14]. Upon acute or chronic stress on the heart, CFs differentiate into *α*-smooth muscle actin (*α*-SMA) expressing myofibroblasts that remodel the cardiac extracellular matrix leading to cardiac fibrosis [15–17]. These differentiated fibroblasts have been also shown to originate mainly from epithelial-to-mesenchymal transition [18] or endothelial-to-mesenchymal transition [19]. More recently, studies demonstrated that resident fibroblasts of epicardial origin, expressing among other progenitor transcription factors, platelet derived growth factor receptor alpha (PDGFR*α*), transcription factor 21 (Tcf21), T-box transcription factor 18 (Tbx18) and Wilms tumour 1 (Wt1), give rise to myofibroblasts in the diseased heart [20–23]. Nevertheless, studies exploring CF implication in gender-dependent cardiac fibrosis with age are still largely lacking.

The objective of this study is to characterize, in a murine aging model of both genders, the patterns of cardiac fibrosis and CF phenotypes. This could lead to reconsider the management of cardiac fibrosis in humans in these conditions and to identify novel therapeutics.

## 2. Materials and Methods

### 2.1. Animals and Study Groups

The present study was approved by the Ethical Committee of Saint Joseph University. Protocols were designed according to the Guiding Principles in the Care and Use of Animals approved by the Council of the American Physiological Society and were in adherence to the *Guide for the Care and Use of Laboratory Animals* published by the US National Institutes of Health (NIH Publication no. 85-23, revised 1996) and according to the European Parliament Directive 2010/63 EU. Animals were housed in s controlled environment at a stable temperature (25°C) and humidity (50 ± 5%) and were exposed to a 12: 12 h light-dark cycle. They were fed ordinary rodent chow, had free access to tap water and were acclimatized for at least one week under these conditions before the start of the study.

The study was conducted in C57BL6/J mice of both sexes; young mice were two months old and aged mice were twenty months old. Mice were randomly divided into 4 groups with a total of 32 animals (*n* = 8 in each group): young male, young female, aged male and aged female.

### 2.2. Transthoracic Echocardiography

Transthoracic echocardiography was conducted using the SonoScape S2V high-resolution color Doppler ultrasound system equipped with a 9 MHz C611 transducer (SonoScape Co., Shenzhen, China) which is specifically designed for mice and rats. Just before sacrifice, mice were anesthetized with isoflurane where 3% was used for induction and 1.5% for maintenance, at a flow of 1 L/min using an EZ-SA800 Anesthesia Single Animal System (E-Z Systems, Pennsylvania, USA). Left ventricular (LV) parasternal long-axis 2D view in *M*-mode was performed at the level of papillary muscle to assess LV wall thicknesses and internal diameters, allowing the calculation of the fractional shortening (FS) and ejection fraction (EF) by the Teicholz method. EF (%) was calculated using the following formula: EF = (EDV − ESV)/EDV ×100; EDV: end-diastolic volume and ESV: end-systolic volume. FS (%) was calculated based on the diameters of the left ventricle: FS = (LVIDd − LVIDs)/LVIDd × 100; LVIDd: end diastolic left ventricular internal diameter and LVIDs: end systolic left ventricular internal diameter. Experiments were done by two independent operators blinded to the conditions.

### 2.3. Histology and Immunofluorescence

Mice were sedated by intraperitoneal injection containing a mixture of ketamine (Ilium, Australia; 75 mg/kg) and xylazine (Interchemie, Holland; 10 mg/kg). Pedal withdrawal reflex was performed to make sure of adequate depth of anesthesia. When animals were completely non-responsive to toe pinching, their hearts were removed, rinsed and perfused with ice-cold Ringer's solution until all blood was removed. Major blood vessels and connective tissue as well as the fat surrounding the heart were discarded, then the heart, with only atria and ventricles, was blotted dry and weighed. Then, it was cut into half through a mid-sagittal plane with one half kept in 10% neutral buffered formalin (4% formaldehyde) and the other embedded in Optimal Cutting Temperature OCT compound. Tibia was cut and removed at the end.

Neutral buffered formalin was used with a pH of 7.0 stabilized by the addition of sodium dihydrogen phosphate monohydrate (NaH_2_PO_4_.H_2_O) and disodium hydrogen phosphate anhydrous (Na_2_HPO_4_). Fixed cardiac tissue was then embedded in paraffin, cut in sections of 4 *μ*m then stained with Masson's Trichrome Masson's trichrome (Sigma-Aldrich, Missouri, USA) for histopathological evaluation. After staining, sections were rinsed in distilled water, dehydrated in ethanol/water baths with decreasing water content, and finally rinsed in xylene before being mounted with a permanent mounting medium. Histological studies were performed by two different pathologists and a scoring system was used to evaluate fibrosis. Representative pictures were at last taken using a VanGuard High-Definition Digital Camera (VEE GEE Scientific, Illinois, USA). Coronary thickness was calculated using ImageJ software. Two sections and two view fields were analyzed in each condition in animals.

Cardiac tissue that was embedded in OCT was submerged and frozen in isopentane (Sigma-Aldrich, Missouri, USA) incubated with dry ice. Cryosections of 4 *μ*m thickness were cut in the same heart location and depth, allowing for a delicate analysis of cardiac fibroblast populations. They were then fixed with the neutral buffer formalin solution for 20 minutes at 4 degrees and antigen retrieval was done by incubating the sections with 2 N HCl for 20 minutes at room temperature. Sections were then incubated for 20 minutes at room temperature with 0.3 M glycine that binds free aldehyde groups that would otherwise bind the primary and secondary antibodies, leading to high background. Permeabilization was achieved with Triton X-100 for 20 minutes at room temperature. Blocking was performed with 10% goat serum and 1% bovine serum albumin diluted in phosphate buffer saline for 1 hour at 37 degrees. Incubation with primary antibodies was done overnight at 4 degrees in blocking buffer; antibodies were: Col I (ab34710; 1/100), Col III (ab7778; 1/100), PDGFR*α* (ab124392; 1/100), *α*-SMA (ab32575; 1/100), Tcf21 (ab32981; 1/100), Wt1 (ab89901; 1/250) (Abcam, Cambridge, USA) and Tbx18 (SAB2102382; 1/100) (Sigma-Aldrich, Missouri, USA). The following day, sections were washed with phosphate buffer saline then incubated with the secondary antibodies for 30 minutes at 37 degrees; antibodies were: Goat anti-rabbit IgG H&L Alexa Fluor 488 and goat anti-rabbit IgG H&L Alexa Fluor 594. The secondary antibodies used were initially pre-adsorbed by passing them through a column matrix containing immobilized serum proteins from the same species the tissue samples originated from i.e. mouse. This extra purification process reduces background by lowering the risk of cross-reactivity between the secondary antibodies and endogenous proteins and immunoglobulins. Finally, sections were mounted with Fluoroshield Mounting Medium containing 4′,6-diamidino-2-phenylindole (DAPI) (Abcam, Cambridge, UK) and pictures were taken using an Axioskop 2 immunofluorescence microscope (Carl Zeiss Microscopy GmbH, Jena, Germany) equipped with a CoolCube 1 CCD camera (MetaSystems, Newton, Massachusetts, USA). Image analysis and quantifications were done using ImageJ. WGA, Col I and Col III quantifications were done by thresholding the acquired pictures, then creating selections of the fluorescent areas. Two sections were analyzed in each condition in animals.

### 2.4. Statistical Analysis

Statistical analysis was performed with the SigmaPlot v11.0 software. All quantitative data are reported as mean ± SEM. Normal distribution of the values was checked by the Kolmogorov-Smirnov test and equal variance was checked by the Levene Median test. Two-way ANOVA tests were performed for the two independent variables, age and sex. To identify which group differences accounted for significant overall ANOVA results, the Holm-Sidak test was used for multiple pairwise comparisons. Significance was set below 0.05 for all analysis.

## 3. Results and Discussion

**Figure 1:**
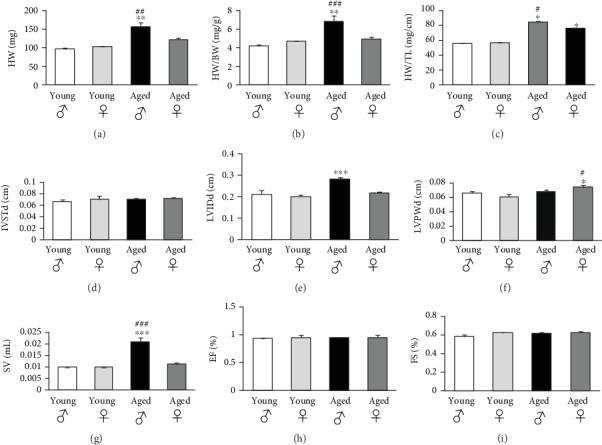
Left ventricular remodeling with age is differentially regulated within genders. (a-i): Representative histograms of heart and echocardiographic parameters in the young and aged mice groups of both sexes. HW: heart weight, BW: body weight, TL: tibia length, IVSTd: end diastolic interventricular septal thickness, LVIDd: end diastolic left ventricular internal diameter, LVPWd: end diastolic left ventricular posterior wall thickness, SV: stroke volume, EF: ejection fraction, FS: fractional shortening. *n* = 8 animals per group. ^*∗*^*p* < 0.05, ^*∗∗*^*p* < 0.01 and ^*∗∗∗*^*p* < 0.001*vs* young male and female, ^*#*^*p* < 0.05, ^*##*^*p* < 0.01 and ^*###*^*p* < 0.001*vs* aged female or male.

Cardiac hypertrophy was present in aged animals of both sexes but was more pronounced in male as evidenced by the increase in heart weights as well as heart weight/body weight and heart weight/tibia length ratios (Figures 1(a)–1(c), respectively). The use of tibia length for normalization has been previously demonstrated to be more reliable than those based on body weight [2]. Therefore, in conditions in which body weight differences may occur, the heart size can be more accurately quantified by relating the heart weight to tibial length. Since body weight represents sexual dimorphism with age in rodent such as in the C57BL6/J mice [24], this might explain the discrepancy seen in the significance levels between heart weight/body weight and heart weight/tibia length ratios. Nevertheless, both ratios demonstrated the same patterns of cardiac mass between the sexes. This cardiac hypertrophy was eccentric with stable septal thickness and increase in left ventricular internal diameter (Figures 1(d) and 1(e)) as opposed to the female mice that developed the concentric type with thicker left posterior wall (Figure 1(f)). Stroke volume reflected chamber dilatation observed only in the male mice hearts (Figure 1(g)). Cardiac hypertrophy has been described in elderly patients to develop differently in women and men; in general, women develop a more concentric form than men with smaller ventricular diameters and less ventricular dilatation [25]. Several studies have demonstrated that with age, male hearts exhibit myocyte loss and appearance of replacement fibrosis. However, this is accompanied by cardiac hypertrophy and increased heart weight since the remaining myocytes undergo volume increase. Female hearts also undergo hypertrophy with less myocyte loss and replacement fibrosis [26]. However, at all ages, the male hearts remain bigger [27]. At a more advanced age, other studies showed that cardiac remodeling in the male progresses toward eccentric hypertrophy which might lead to heart failure, whereas concentric cardiac hypertrophy and preserved function remain in the female [28–30]. Whereas cross-sectional studies have shown both increases and decreases in cardiac mass with age, a longitudinal observation of a large cohort of asymptomatic individuals who were free of clinical cardiovascular disease at baseline, showed a longitudinal increase in cardiac mass with age in men [31]. In our murine model, the males had larger left ventricular cavities than the females, whereas the latter had thicker posterior walls; but, the bigger and most importantly the heavier male hearts show compensatory hypertrophy that explains the higher stroke volume. Yet again, their hypertrophy may be at a higher risk of decompensation as shown in literature and as demonstrated by the important presence of replacement fibrosis in our model.

Cardiac function evaluation i.e. ejection fraction (EF) and fractional shortening (FS) remained stable with age (Figures 1(h) and 1(i)). These features are similar to some of the attributes of HFpEF in humans [32–35]. Geroscience has undoubtedly shown that HFpEF constitutes the most common type of heart failure in the elderly and mainly in the female [1, 2, 9]. The heart of HFpEF patients exhibits structural alterations including cardiac hypertrophy, interstitial fibrosis and coronary capillary rarefaction. These alterations may modify heart dynamics such as increase in left ventricular passive stiffness, impairment in relaxation, elevation in left ventricular end-diastolic pressure and enlargement of left atrium due to increased filling pressures [36]. In our murine aging model, the lack of left ventricular dynamics assessment was a limitation for further substantiating HFpEF installation.

**Figure 2:**
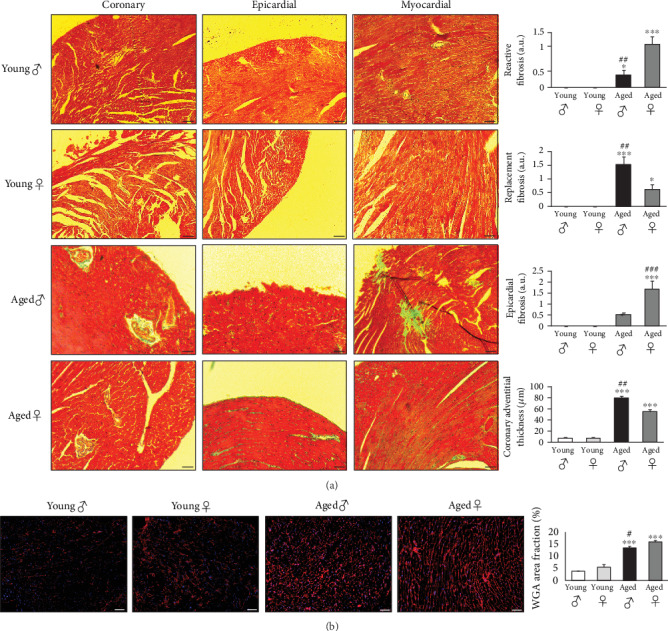
Gender-dependent patterns of cardiac fibrosis with age. (a): Perivascular coronary, epicardial and myocardial histological sections stained in Masson's trichrome obtained from young and aged mice of both sexes, as well as quantifications of interstitial reactive, replacement and epicardial fibrosis, and coronary adventitial thickness. The green stains represent the fibrotic areas. (b): Representative images and quantifications of immunofluorescence staining (594 nm) for whecat germ agglutinin (WGA) in the hearts from young and aged mice of both sexes. Nuclei are stained blue with DAPI. Scale bars: 100 *μ*m in (a) and 50 *μ*m in (b). Magnifications: x100 in (a) and x200 in (b). *n* = 8 animals per group, *n* = 3 fields of view per condition. ^*∗*^*p* < 0.05 and ^*∗∗∗*^*p* < 0.001*vs* young male and female, ^*#*^*p* < 0.05, ^*##*^*p* < 0.01 and ^*###*^*p* < 0.001*vs* aged female or male.

A thorough histological analysis of cardiac sections stained with Masson's trichrome was conducted to study the location and extent of cardiac fibrosis. Perivascular, sub-epicardial and interstitial regions of the left ventricle were examined for total collagen deposition; perivascular fibrosis was defined as collagen accumulation in the adventitia of coronary arteries. Young mice hearts of both sexes presented no signs of epicardial or interstitial fibrosis and had coronary vessels with thin adventitia (Figure 2(a)). With age, male and female mice showed distinct patterns of cardiac fibrosis. Interstitial reactive and epicardial fibrosis were prominent in the female heart whereas thicker adventitia with scattered myocardial necrotic regions, filled with replacement fibrosis, were features of the male heart (Figure 2(a)). These results were further confirmed by wheat germ agglutinin staining that revealed an important expansion of the cardiac interstitium in the aged female hearts (Figure 2(b)). According to the literature, only cardiac imaging have shown that age is an important independent predictor of cardiac extracellular volume [5, 7, 12]. Also, aged male rat hearts were shown to be larger, thinner and more fibrotic than the female's [37]. Conversely, reactive interstitial fibrosis was associated with left ventricular hypertrophy more commonly in women in imaging studies [7, 38]. This might arise from the fact that the male heart is more prone to undergo extended myocyte apoptosis with age compared to the female's [39]. Our study is the first to demonstrate in mice, on a cellular level, the presence of differential age-related cardiac fibrosis patterns between the male and the female.

**Figure 3:**
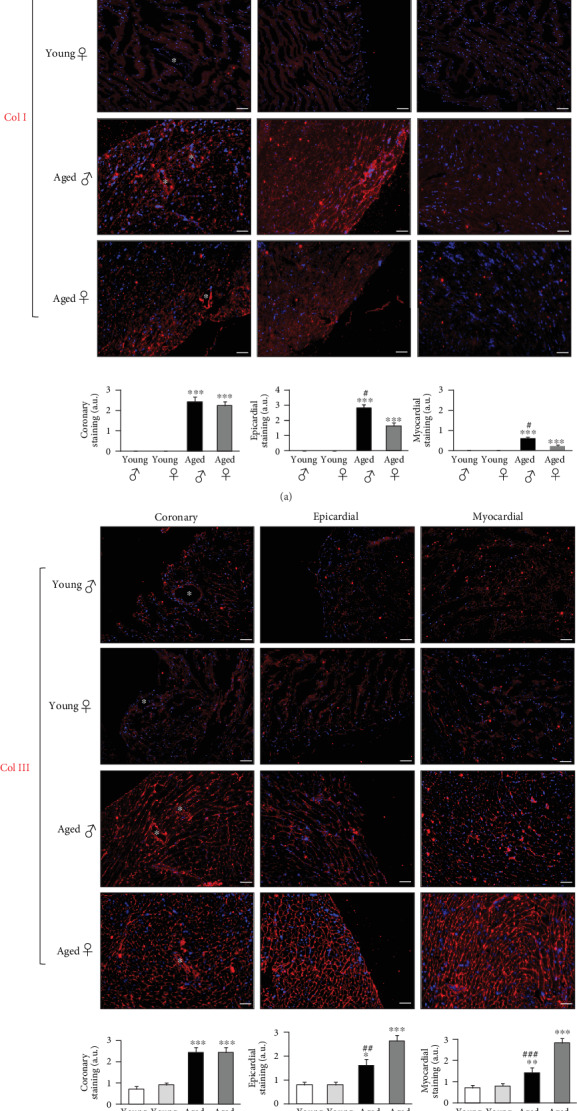
Cardiac collagen displays gender-dependent profiles with age. (a, b): Perivascular coronary, epicardial and myocardial immunofluorescence staining (594 nm) for collagen I (Col I) and collagen III (Col III) of histological sections obtained from young and aged mice of both sexes, as well as quantifications of the respective stained zones. Nuclei are stained blue with DAPI. White asterisks indicate coronary vessels. Scale bars: 50 *μ*m. Magnifications: x200. *n* = 8 animals per group, *n* = 3 fields of view per condition. ^*∗*^*p* < 0.05, ^*∗∗*^*p* < 0.01 and ^*∗∗∗*^*p* < 0.001*vs* young male and female, ^*#*^*p* < 0.05, ^*##*^*p* < 0.01 and ^*###*^*p* < 0.001*vs* aged female or male.

Distribution of the main collagen isoforms within the heart displayed age and gender dependencies. Collagen I and III were present at low levels in the young mice hearts of both sexes and drastically increased with age (Figures 3(a) and 3(b)). Epicardial and myocardial collagen I staining were higher in the aged male heart as compared to the female's (Figure 3(a)). Oppositely, epicardial and myocardial collagen III staining were higher in the aged female heart (Figure 3(b)). No dissimilarities were seen on coronary staining for both collagen isoforms between the two groups of mice (Figures 3(a) and 3(b)). During disease in humans, such as in aortic stenosis, the male heart has been shown to undergo changes in collagen architecture with higher collagen I and III compared to women's that might account for the depressed cardiac function [11, 40]. Moreover, it has been reported that increased cardiac content of collagen I produces maladaptive remodeling due to pressure overload, whereas increased levels of collagen III leads to improved cardiac function [41, 42]. When it comes to cardiac aging, collagen has been known to increase in the human heart through an imbalance in its turnover [3] along with a change in its characteristics [43] leading to left ventricular stiffness, impaired relaxation and increase in filling cardiac cavities pressure, hallmarks of HFpEF. Whereas some studies described the increase in cardiac collagen I, others reported the increase in collagen III [8, 44, 45]. These discrepancies might rise from the heterogeneous studied subjects with no distinction between the sexes. Here, we give a clearer evidence of the distinct collagen phenotypes in the aged male and female mice hearts. This can be explained by distinct regulations of collagen I and III by female hormones [46].

**Figure 4:**
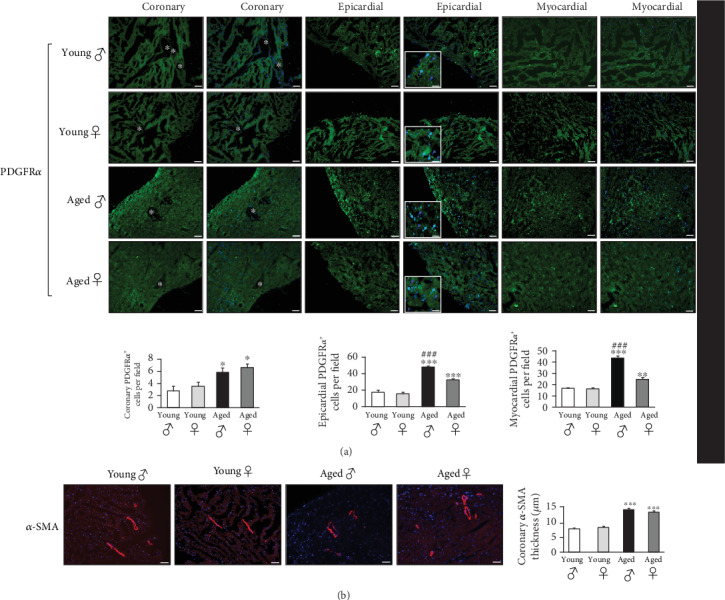
Differential gender-dependent distribution of fibroblast populations in the young and aged hearts. (a) Perivascular coronary, epicardial and myocardial immunofluorescence staining (488 nm) for PDGFR*α* of histological sections obtained from young and aged mice of both sexes, as well as quantifications of the respective stained zones. Dark blue arrows indicate cardiomyocytes nuclei stained with DAPI, whereas light blue arrows indicate co-localization of DAPI and PDGFR*α* in cardiac fibroblasts. White asterisks indicate coronary vessels. (b) Immunofluorescence staining (594 nm) for *α*-SMA of histological sections obtained from young and aged mice hearts of both sexes, as well as quantifications of the respective stained zones. Nuclei are stained blue with DAPI. Scale bars: 50 *μ*m. Magnifications: x200. *n =8* animals per group, *n* = 3 fields of view per condition. ^*∗*^*p* < 0.05, ^*∗∗*^*p* < 0.01 and ^*∗∗∗*^*p* < 0.001*vs* young male and female, ^*###*^*p* < 0.001*vs* aged female or male.

**Figure 5:**
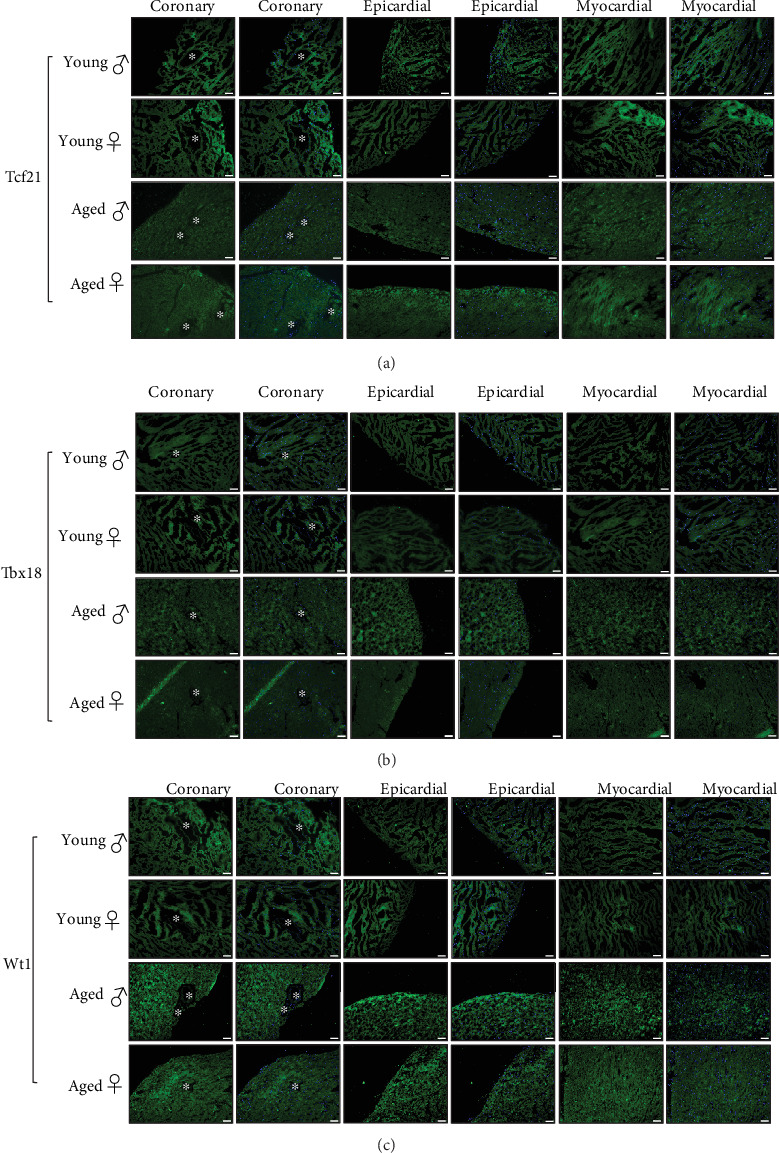
Non-epicardial origin of cardiac fibroblasts in the young and aged hearts. (a-c) Perivascular coronary, epicardial and myocardial immunofluorescence staining (488 nm) for Tcf21, Tbx18 and Wt1 of histological sections obtained from young and aged mice of both sexes. Nuclei are stained blue with DAPI. White asterisks indicate coronary vessels. Scale bars: 50 *μ*m. Magnifications: x200. *n* = 8 animals per group, *n* = 3 fields of view per condition. ^*∗*^*p* < 0.05*vs* young male and female, ^*#*^*p* < 0.05*vs* aged female or male.

The implication of CFs, as the main contributors to cardiac fibrosis, was finally studied by analyzing CFs lineage and distribution in the different mice groups. A significant age-related increase in PDGFR*α* expression was seen in all of the studied heart regions (Figure 4(a)). These PDGFR*α*^+^ cells were predominantly present in the epicardial and myocardial regions of the male heart as compared to the female's (Figure 4(a)). This large expansion of PDGFR*α*+ CFs in the male heart states that resident CFs could play a major role in gender-related cardiac fibrosis with age, since PDGFR*α* has been shown as a selective marker of resident CFs through development and in the adult's heart [47, 48]. The myofibroblast marker *α*-SMA was only present around coronary vessels with higher expression in the aged hearts (Figure 4(b)). Nonetheless, epicardial progenitor transcription factors, Tcf21, Tbx18 and Wt1 were all undetectable in the young and aged mice hearts of both sexes (Figures 5(a)–5(c), respectively). Age-associated cardiac fibrosis has been linked to the dysregulation of resident mesenchymal fibroblasts in the myocardium [49–52]. Intriguingly, this total absence of epicardial markers in these resident CFs might be explained by the fact that cell markers differ according to the category of resident CFs and their degree of differentiation [21, 53, 54] and that distinct fibrogenic mechanisms exist depending on the underlying pathology [55]. Besides, in a previous study, we showed the absence of these markers in the young mice hearts and their appearance only after the induction of disease i.e. cardiac hypertrophy and fibrosis [56]. To our knowledge, this is the first study to demonstrate that patterns and phenotypes of CFs in the aged mice hearts are gender-dependent.

Cardiac fibrosis observed in male and female mice with age did not lead to significant declines in heart function. In our study, this might be related to our model whereby old animals had twenty months. However, based on the presence of replacement fibrosis along with the rigid collagen I and the higher number of PDGFR*α*^+^ CFs in these old male hearts, one might postulate that at a more advanced age, i.e. very old, this could lead to the establishment of functional divergences between the sexes with a decline in cardiac function in the male.

## 4. Conclusions

**Figure 6:**
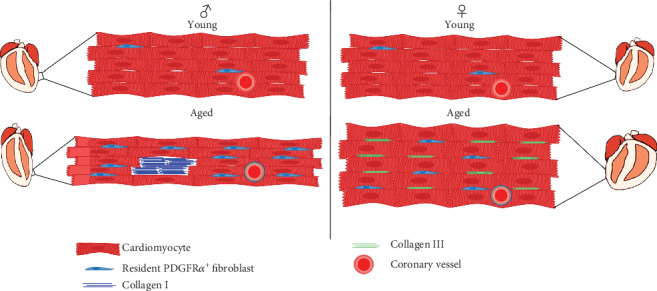
Schematic representation of gender-dependent cardiac fibrosis and CF populations with age. Eccentric cardiac hypertrophy is present in the aged male whereas concentric cardiac hypertrophy is present in the female. Replacement myocardial fibrosis was evidenced in the aged male heart over reactive myocardial one in the female. Collagen I is predominant in the aged male hearts whereas collagen III is the main component in the female's. CFs in the aged male heart were mainly recruited from resident PDGFR*α*^+^ populations. CF: cardiac fibroblast.

Our results show that gender-related cardiac fibrosis and CF populations in mice display particular and different patterns with age (Figure 6). These findings constitute a step forward to better understanding and management of cardiac fibrosis in the elderly in humans and can help paving a way toward novel therapeutic targets.

## Data Availability

The majority of the data can be found in the manuscript, and further data used to support the findings of this study are available from the corresponding author upon reasonable request.
